# Relationship between esophageal squamous cell carcinoma risk and alcohol‐related ALDH2 and ADH1B polymorphisms: Evidence from a meta‐analysis and Mendelian randomization analysis

**DOI:** 10.1002/cam4.6610

**Published:** 2023-10-05

**Authors:** Biao Zhang, Yu‐Hui Peng, Yun Luo, Chao‐Qun Hong, Yi‐Wei Lin, Yu‐Ling Zhang, Yi‐Wei Xu, Xue‐Fen Su, Fang‐Cai Wu

**Affiliations:** ^1^ Department of Clinical Laboratory Medicine Cancer Hospital of Shantou University Medical College Shantou China; ^2^ Department of Preventive Medicine Bin Hai Wan Central Hospital of Dongguan Dongguan China; ^3^ Esophageal Cancer Prevention and Control Research Center The Cancer Hospital of Shantou University Medical College Shantou China; ^4^ Precision Medicine Research Center Shantou University Medical College Shantou China; ^5^ Yongchuan Hospital Affiliated to Chongqing Medical University Chongqing China; ^6^ Research Institute of Clinical Pharmacy, Shantou University Medical College Shantou China; ^7^ Department of Radiation Oncology Cancer Hospital of Shantou University Medical College Shantou China

**Keywords:** ADH1B, ALDH2, epidemiological evidence, esophageal squamous cell carcinoma, meta‐analysis

## Abstract

**Background:**

Previous studies have shown that ALDH2 and ADH1B genes may be associated with alcohol metabolism and the risk of esophageal squamous cell carcinoma (ESCC), with inconsistent results. This meta‐analysis aimed at comprehensively assessing the associations between ALDH2 and ADH1B polymorphisms and the risk of ESCC to synthesize and clarify the evidence.

**Methods:**

We calculated summary estimates of the associations between four genetic variants (rs671 and rs674 in ALDH2, and rs1229984 and rs1042026 in ADH1B) and the ESCC risk across 23 publications in the additive model and allelic model. Venice criteria, Bayesian false discovery probability (BFDP), and false‐positive reporting probability (FPRP) were used to assess the strength of epidemiological evidence. Heterogeneity among studies was evaluated by using the Higgin's *I* ^2^ statistic, and publication bias was assessed by using funnel plots and Begg's test. A Mendelian randomization (MR) analysis was performed to determine the causal association between alcohol intake and esophageal cancer risk. Data from the HaploReg v4.1 and PolyPhen‐2 were analyzed for functional annotations.

**Results:**

Of the four genetic variants, rs671 of ALDH2 was associated with a significantly reduced risk of ESCC (OR: 0.60, 95% CI: 0.50–0.73), whereas rs1229984 of ADH1B was associated with a significantly increased risk (2.50, 95% CI: 1.70–3.69) in the additive model. In the allelic model, the variant rs1229984 of ADH1B also increased the risk of ESCC (OR: 1.50; 95% CI: 1.21–1.87). The result for the variant rs671 was considered as strong epidemiological evidence. Functional annotations identified that the four variants were related to the enhancer histone marks and motif changes. The other two variants were not associated with the ESCC risk (rs674 of ALDH2 OR: 1.22, 95% CI: 0.71–2.12; rs1042026 of ADH1B OR: 1.28, 95% CI: 0.52–3.14) in the additive model. The MR analysis did not find a causal effect of alcohol on the esophageal cancer risk.

**Conclusions:**

The results showed that ADH1B rs1229984 was significantly associated with an increased the risk of ESCC.

## 1 INTRODUCTION

Globally, esophageal cancer ranks the seventh in cancer incidence and the sixth in cancer mortality, with approximately 90% of the pathological types being esophageal squamous cell carcinoma (ESCC).^1^ With 604,100 incident cases of esophageal cancer and 544,076 deaths in 2020,^2^ ESCC caused a huge medical and economic burden on a global scale. Epidemiological studies have shown that smoking, alcohol drinking, living habits, lifestyles, and income are the factors that may influence the risk of ESCC.^1^, ^3^ As for alcohol, acetaldehyde plays an important role in the first product of ethanol metabolism and is a class I carcinogen with strong carcinogenic effect.^4^, ^5^ Ethanol was primarily metabolized to acetaldehyde in the liver by alcohol dehydrogenases (ADHs), including ADH1B, and then to acetate by aldehyde dehydrogenase 2 (ALDH2).^6^

The association between alcohol consumption and the risk of ESCC exhibits regional differences. Regions with low incidence of ESCC showed stronger association with alcohol consumption than those with high incidence.^7^, ^8^, ^9^ This difference may be attributed to different populations/races living in various regions and/or other related factors that require further research,^10^ for instance, differences in alcohol metabolism among populations. Studies have shown that ALDH2 and ADH1B are associated with reduced rates of alcohol dependence.^11^ The variants of the ALDH2 and ADH1B genes are mainly ALDH2*1 and ADH1B*2, which metabolize alcohol more rapidly and lead to excessive accumulation of acetaldehyde.^12^, ^13^, ^14^, ^15^ Excessive acetaldehyde can cause a heightened response to alcohol.^16^ Furthermore, the frequency of ALDH2 and ADH1B polymorphisms varies in different populations or regions.^17^ This suggests that individual gene polymorphisms may cause differential genetic susceptibilities to ESCC.

During the past two decades, studies have been carried out to explore the ALDH2 and ADH1B genetic variants and the risk of ESCC. A previous meta‐analysis that investigated the associations between ALDH2 and ADH1B polymorphisms and ESCC risk was published in 2010.^18^ The results indicated that ADH1B rs1229984 and ALDH2 rs671 were susceptible loci for ESCC in the Chinese population. Since then, 11 new relevant studies have been published in recent years with inconsistent results. In addition, several GWAS reports identified multiple genetic variants in ALDH2 and ADH1B as susceptibility loci in Chinese.^19^ Furthermore, the previous meta‐analysis did not use Venice criteria, false‐positive reporting probability (FPRP), and Bayesian false discovery probability (BFDP) to assess the epidemiological evidence of the ALDH2 and ADH1B polymorphisms and the ESCC risk. Therefore, we included all published studies retrieved from the literature to perform an updated comprehensive meta‐analysis to assess the associations between the ALDH2 and ADH1B polymorphisms and the ESCC risk. The epidemiological evidence of the associations was evaluated by using the Venice criteria, FPRP, and BFDP.

## 2 MATERIALS AND METHODS

This systematic review and meta‐analysis was performed according to the PRISMA guidelines.^20^ In addition, we registered a protocol with the International Prospective Register of Systematic Reviews–PROSPERO (Registration No. CRD42022357068).

### 2.1 Literature search and selection criteria

We searched the literature in PubMed and Embase using the terms “((esophageal squamous cell carcinoma) AND (ALDH2)) OR ((esophageal squamous cell carcinoma) AND (ADH1B))”. All relevant articles published from database inception to August 2022 were included in the literature search. Included publications conformed to the following criteria: (1) The study design was a case–control study, or a genome‐wide association study (GWAS) conducted among human populations; (2) all ESCC cases were confirmed by pathological diagnosis; (3) the articles provided sufficient data on odds ratios (OR) and 95% confidence intervals (CIs) in additive or allelic model; and (4) it was published in peer‐reviewed journals in English. The exclusion criteria were as follows: (1) duplicate literature; (2) detailed information on gene variants and/or ORs was unavailable; and (3) the study did not assess the association between genetic variants and susceptibility to ESCC.

### 2.2 Data extraction

Data were extracted by two researchers (Zhang B. and Luo Y.) using a standard data extraction form. The extracted information included: the first author, publication year, study design, country where the study was conducted, population ethnicity, gene and variant, the number of samples in the case and control groups, the OR and corresponding 95% CI in an allelic model or additive model. All disagreements were discussed, and consensus was obtained.

### 2.3 Statistical analysis

Stata 12.0 was used to analyze all the data. Meta‐analyses were conducted if at least three studies reported the association between the ALDH2 and ADH1B genetic variants and the ESCC risk in the additive model (AA vs. GG) and allelic model (A vs. G). The additive model is widely used as a conservative choice between the recessive and the dominant model, and most of the included studies in the meta‐analysis provided the data of OR and 95% CI in the additive and allelic models. The summary odds ratios (ORs) or 95% confidence intervals (95% CIs) were calculated. The OR was converted to log (OR). Because the covariates adjusted were different in various studies, we used the crude OR to perform the meta‐analysis. Higgin's *I* ^2^ statistic was used to quantitatively assess the level of heterogeneity. An *I* ^2^ statistic of 0% indicates no observed heterogeneity, whereas *I* ^2^ statistics less than 25% suggest mild heterogeneity, between 25% and 50% indicating moderate heterogeneity, and greater than 50% demonstrating high heterogeneity.^21^, ^22^ The *p*‐value of Cochran's *Q* less than 0.1 indicates that the heterogeneity is statistically significant. If the *I* ^2^ is less than 50%, a fixed‐effect model is applied. A random‐effect model was used, when the *I* ^2^ is greater than 50%. Stratified analyses and meta‐regression were also performed to explore the potential sources of heterogeneity. Sensitivity analysis was used to assess the stability of results. Funnel plots, Begg's test, and Egger's test were deployed to analyze whether a publication bias was indicated.^23^ All tests were two‐sided, and *p* < 0.05 indicated statistical significance unless otherwise stated.

### 2.4 Assessment of epidemiological reliability

The Venice criteria, proposed by the Human Genome Epidemiology Network (HuGENet) Working Group, were used to assess the strength of epidemiological evidence.^24^ The evidence of each significant association derived from the meta‐analysis was graded according to three standards: the amount of evidence, replication of association, and protection from bias. For the amount of evidence, the sum of the number of minor alleles among cases and controls of more than 1000 in the meta‐analysis was defined as A, between 100 and 1000 classified as B, and less than 100 defined as C. For the replication of association, an A grade indicates that the heterogeneity statistic *I* ^2^ was less than 25%, B means the *I* ^2^ value is between 25% and 50%, and C represents the *I* ^2^ greater than 50%. Moreover, a significant association might be obtained due to incorrectly classified genotypes and/or publication bias. Therefore, if there was no observable bias that could affect the results, an A grade was given. The B grade suggests no apparent bias, but there was considerable missing information, and the C grade means there was significant bias that could affect the results. Ultimately, the epidemiological reliability for a significant genetic association was strong when all criterion grades were A and weak if any criterion grade was C. The other combinations indicated that the evidence was moderate.

The false‐positive reporting probability (FPRP) was calculated to determine whether a significant association could be considered as a false‐positive result. The prior probability was set to 0.05 to detect an OR of 1.5.^25^ When FPRP was <0.2, we considered the association as true. If a significant association based on the FPRP analysis was true, the cumulative evidence was upgraded from weak to moderate and from moderate to strong. Bayesian false discovery probability (BFDP) is a new statistical method based on logistic regression models and does not depend on statistical power. When the BFDP value is less than 0.8, the association is considered as true.^26^ Although the false‐positive reporting probability and the Bayesian false discovery probability are similar, the FPRP is a traditional and commonly used method to assess the false‐positive probability if significant associations are found, and the BFDP is a new method that does not depend on statistical power. Moreover, using both methods could verify and further improve the credibility of the results. If inconsistent results were found between FPRP and BDFP analyses, we used the result of FPRP to judge the authenticity of a significant association.

### 2.5 Mendelian randomization analysis

Mendelian randomization analysis was performed by using the inverse variance weighted (IVW) to explore the causal relationship between alcohol (exposure) and esophageal cancer (outcome) through the TwoSampleMR package in R version 4.0.2. Because the IEU Open GWAS only had esophageal cancer data, we analyzed the casual association between alcohol and esophageal cancer risk. The genetic instrument was retrieved from the GWAS data of the UK Biobank study including 456,382 samples. The genetic instrument should meet the following criteria: (1) There was a significant correlation between SNPs and alcohol at genome‐wide significance (*p* < 5 × 10^−8^); and (2) SNPs are independent of each other (linkage disequilibrium (LD) in the range of 10 Mb *r* ^2^ < 0.05). In addition, we evaluated the presence of horizontal pleiotropy and heterogeneity using MR‐Egger intercept and *Q* test.

## 3 RESULTS

### 3.1 Characteristics of included studies

**FIGURE 1:**
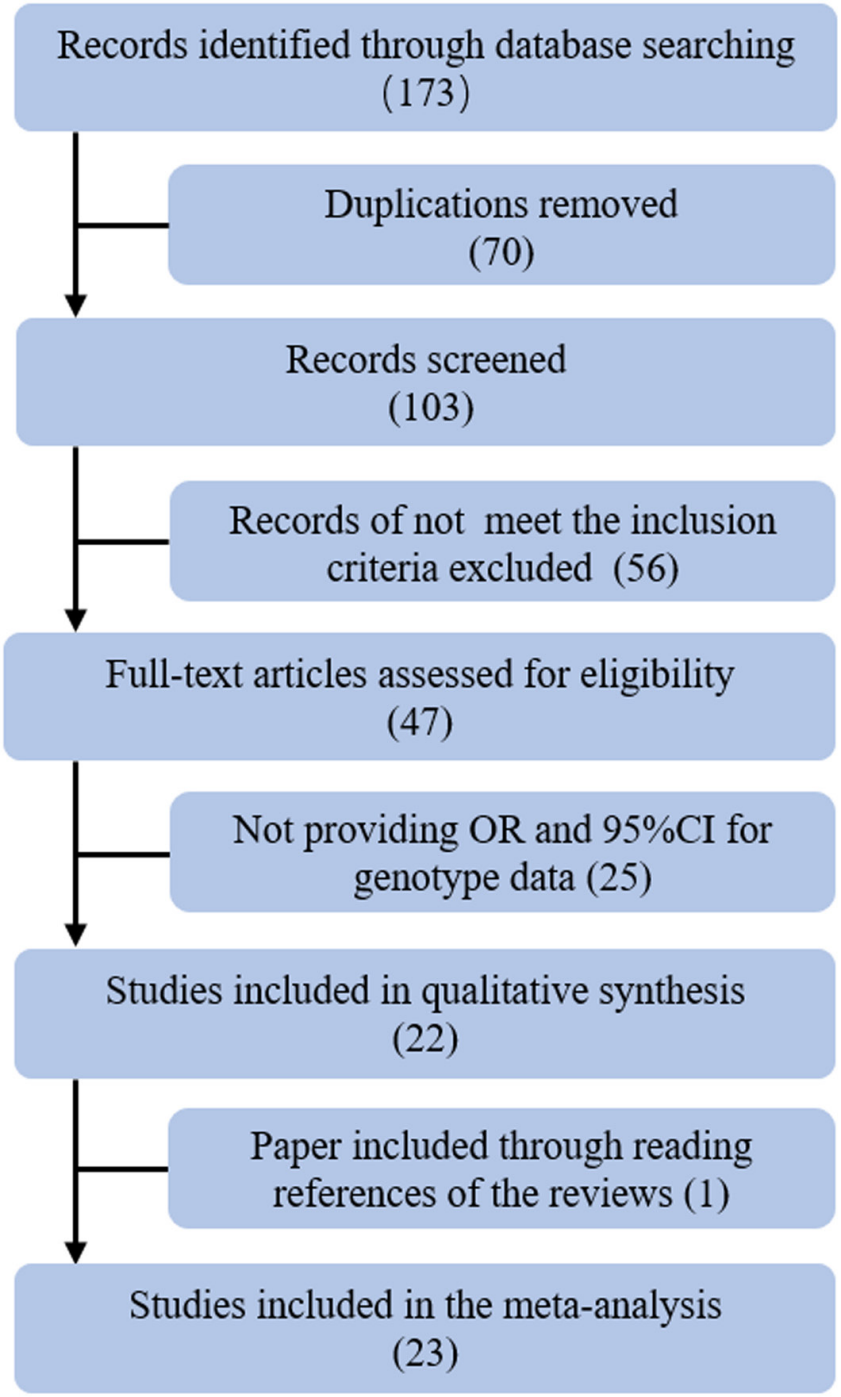
Flow chart of literature search based on a two‐step search strategy.

The process of literature search and article inclusion is shown in Figure 1. One seventy‐three articles were found during the literature search, of which 70 were duplicates and 56 were not related to ALDH2 or ADH1B gene polymorphisms. Among the remaining 47 articles, 25 did not provide an OR and 95% CI. Therefore, 22 articles were left. An additional study was retrieved through reference review. Hence, 23 articles were included in the analysis.

The details of the 23 included papers are shown in Table 1. Most of the studies were published before 2019. Twenty‐one were case–control and two were genome‐wide association (GWAS) studies. Twenty‐one studies were conducted among Asian population, and the other two were among Africans. The number of cases varied from 37 to 2098 and the number of controls varied from 31 to 2763. The genetic variants of ADH1B included rs1042026, rs17033, rs1159918, and rs1229984, and the genetic polymorphisms of ALDH2 included rs671, rs674, rs4767364, and rs886205. The OR values and the corresponding 95% CIs for the allelic and additive models are shown in Table 1.

**TABLE 1:** Characteristics of the included studies.

Study, year	Study design	Country	Race	Gene	Variant	No. of Cases	No. of Controls	Allelic model	Additive model
								OR (95%CI)	
Cai, 2006	Population‐based case–control study	China	Asian	ALDH2	rs671	81	162	–	1.72 (0.85–3.48)
Cui, 2009	Genome‐wide association study	Japan	Asian	ADH1B	rs1042026	1067	2763	1.83 (1.57–2.14)	3.48 (2.33–5.20)
				ADH1B	rs1159918			1.88 (1.59–2.21)	4.02 (2.54–6.37)
				ADH1B	rs1229984			1.79 (1.69–1.88)	4.10 (3.24–5.18)
				ALDH2	rs671			1.67 (1.58–1.76)	0.47 (0.28–0.78)
Gao, 2013	Hospital‐based case–control study	China	Asian	ADH1B	rs1229984	2098	2263	1.24 (1.13–1.36)	1.54 (1.25–1.89)
				ALDH2	rs671			0.83 (0.75–0.91)	0.64 (0.51–0.82)
Guo, 2013	Hospital‐based case–control study	China	Asian	ADH1B	rs1229984	80	480	–	1.46 (0.71–2.59)
Li, 2011	Population‐based case–control study	China	Asian	ALDH2	rs674	226	246	–	3.38 (1.64–6.95)
Liu, 2017	Hospital‐based case–control study	China	Asian	ALDH2	rs671	37	31	–	0.78 (0.12–5.01)
				ALDH2	rs4767364	37	31	–	1.21 (0.16–9.08)
Suo, 2019	Population‐based case–control study	China	Asian	ADH1B	rs1042026	1448	1992	–	0.54 (0.39–0.76)
				ADH1B	rs17033			–	2.60 (1.40–4.97)
				ALDH2	rs671			–	0.58 (0.38–0.85)
Wang, 2014	Hospital‐based case–control study	China	Asian	ADH1B	rs1042026	615	537	–	5.40 (3.19–9.11)
Wang, 2011	Hospital‐based case–control study	China	Asian	ADH1B	‐	81	162	1.51 (0.93–2.37)	–
				ALDH2	‐			1.73 (0.81–2.79)	0.65 (0.22–2.18)
Yao, 2018	genome‐wide association study	China	Asian	ADH1B	rs1042026	1033	2285	1.02 (0.94,1.11)	–
				ALDH2	rs671			0.56 (0.46,0.68)	–
				ALDH2	rs4767364			1.06 (0.93,1.21)	–
Ye, 2014	Hospital‐based case–control study	China	Asian	ADH1B	rs1229984	1001	1391	–	2.81 (2.18–3.62)
Chen, 2006	Hospital‐based case–control study	China	Asian	ADH1B	rs1229984	330	592	–	5.65 (3.67–8.69)
				ALDH2	rs674	330	592	–	0.78 (0.38–1.62)
Kagemoto, 2016	Population‐based case–control study	Japan	Asian	ALDH2	rs671	117	1125	2.02(1.3–3.14)	–
				ADH1B	rs1229984	117	1125	2.05(1.32–3.2)	–
Yokoyama, 2006	Hospital‐based case–control study	Japan	Asian	ADH1B		52	412	–	0.48 (0.18–1.31)
				ALDH2		52	412	–	1.49 (0.47–4.78)
Gu, 2012	Hospital‐based case–control study	China	Asian	ADH1B	rs1229984	380	380	–	2.35 (1.40–3.93)
				ALDH2	rs886205			0.58 (0.34–0.87)	–
Bye, 2011	Population‐based case–control study	South Africa	African	ADH1B	rs1229984	201	427	0.52 (0.32–0.86)	–
				ALDH2	rs886205			0.70 (0.55–0.89)	–
Tanaka, 2012	Hospital‐based case–control study	Japan	Asian	ADH1B	rs1229984	168	1083	1.82 (1.63–2.03)	4.08 (3.27–5.09)
				ALDH2	rs671	742	820	1.78 (1.60–1.98)	3.54 (3.04–4.14)
Yokoyama, 2002	Hospital‐based case–control study	Japan	Asian	ALDH2	–	234	634	–	7.83 (1.33–46.08)
Yokoyama, 2003	Population‐based case–control study	Japan	Asian	ALDH2	–	65	206	–	1.07 (0.95–1.22)
Ma, 2010	Hospital‐based case–control study	China	Asian	ALDH2	rs886205	128	326	1.63 (1.16–2.28)	–
Chen, 2019	Population‐based case–control study	South Africa	African	ALDH2	rs4767364	591	852	1.17 (0.95–1.44)	‐
Ding, 2009	Population‐based case–control study	China	Asian	ALDH2	rs671	191	221	2.15 (1.57–2.95)	5.69 (2.51–12.18)
Yang, 2007	Hospital‐based case–control study	China	Asian	ALDH2	–	191	198	1.05 (0.76–1.44)	0.26 (0.06–1.09)

Abbreviations: OR, odds ratio; CL, confidence interval; −, data not available.

### 3.2 Principal meta‐analyses

**FIGURE 2:**
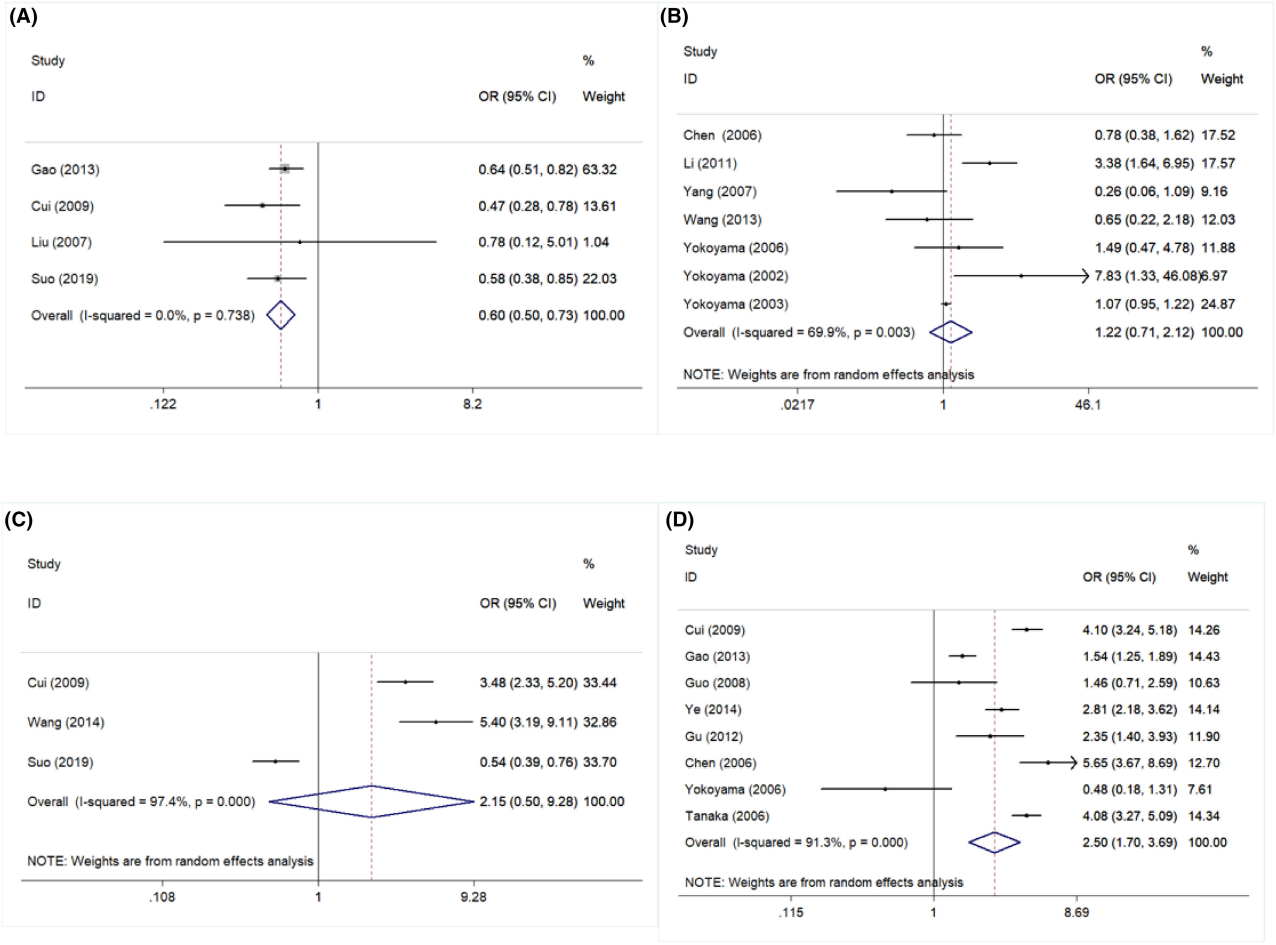
Forest plots of the associations between four genetic variants and esophageal squamous cell carcinoma risk. (A) ALDH2 rs671, (B) ALDH2 rs674, (C) ADH1B rs1042026, and (D) ADH1B rs1229984.

Because only four (rs671 and rs674 of gene ALDH2, rs1229984, and rs1042026 of gene ADH1B) out of the eight genetic polymorphisms had been studied in at least three articles, the four genetic variants were evaluated for their associations with the ESCC risk. The meta‐analyses were performed for these four variants to calculate the summary estimates using results from the additive models (Table 2). The variant rs671 of ALDH2 was associated with a significantly decreased risk of ESCC (OR: 0.60; 95% CI: 0.50–0.73). The variant rs1229984 of ADH1B was associated with a significantly increased risk of ESCC (OR: 2.5; 95% CI: 1.70–3.69). The other two variants, rs674 of ALDH2 (OR: 1.22; 95% CI: 0.71–2.12) and rs1042026 of ADH1B (OR: 2.15; 95% CI: 0.50–9.28) also showed an increased risk with ESCC. However, the association between two variants, rs674 of ALDH2 and rs1042026 of ADH1B, and ESCC risk did not reach statistical significance (Figure 2). As shown in Figure S1, in the allelic model, we found that the variant rs1229984 of ADH1B increased the risk of ESCC (OR: 1.50; 95% CI: 1.21–1.87), but ALDH2 rs671 was not associated with ESCC risk significantly (OR: 1.33; 95% CI: 0.92–1.92).

**TABLE 2:** Genetic variants with a significant association with esophageal squamous cell carcinoma.

Gene	Variant	Study	Allele^a^	No. of Cases	No. of Controls	Genetic model	OR (95% CI)	*I* ^2^ (%)	*p*‐Value	Venice criteria	FPRP OR1.5	BFDP	Level of evidence
											0.05	0.05	
ALDH2	rs671	4	G > A	4655	6941	Additive model	0.60 (0.50–0.73)	0	0.738	BAA	0	0	Strong
ADH1B	rs1229984	8	T > C	5176	7364	Additive model	2.5 (1.70–3.69)	91.3	0	ACA	0.015	0.098	Moderate
ADH1B	rs1042026	3	T > A	4581	5365	Additive model	2.15 (0.50–9.28)	97.4	0	BCA	0.946	0.953	Weak
ALDH2	rs674	7	G > A	1179	2471	Additive model	1.22 (0.71–2.12)	69.9	0.003	BCA	0.922	0.956	Weak
ADH1B	rs1229984	8	T > C	5176	7364	Allelic model	1.50 (1.21–1.87)	93	0	ACA	0.012	0.209	Moderate
ALDH2	rs671	6	G > A	5248	9477	Allelic model	1.33 (0.92–1.92)	98.1	0	ACA	0.767	0.937	Weak

*Note*: Venice criteria grades are for the amount of evidence, replication of the association, and protection from bias: A (high), B (moderate), C (weak) reliability.

Abbreviations: *, one variant was not scored due to the amount of evidence; BFDP, Bayesian false‐discovery probability; C, cytosine; G, guanine; A, adenine; T, thymine; CI, confidence interval; FPRP: false positive report probability; OR, odds ratio.

^a^ Minor alleles vs. major alleles.

In addition, a significant interaction between smoking and the rs1229984 risk allele with ESCC was observed (Table 3). Smokers with rs1229984 risk allele had further increased risk of ESCC compared with nonsmokers. The interaction between smoking and rs671 did not reach statistical significance.

**TABLE 3:** Association of gene–environment interaction in esophageal squamous cell carcinoma in additive model.

Gene	Variants	Environmental factors	No. of studies	Model	Pooled OR (95%CL)	*p* for interaction	Heterogeneity	
							*I* ^2^ (%)	*p‐*Value
ADH1B	rs1229984	Nonsmoker	4	Random	1.39 (1.21–1.59)	<0.001	76.5	0.005
		Smoker	4	Random	1.52 (1.36–1.70)		95.8	0
ALDH2	rs671	Nonsmoker	3	Random	0.95 (0.84–1.07)	0.103	91.8	0
		Smoker	3	Random	2.28 (0.65–8.02)		99.0	0

### 3.3 Heterogeneity, sensitivity analysis and publication bias

**FIGURE 3:**
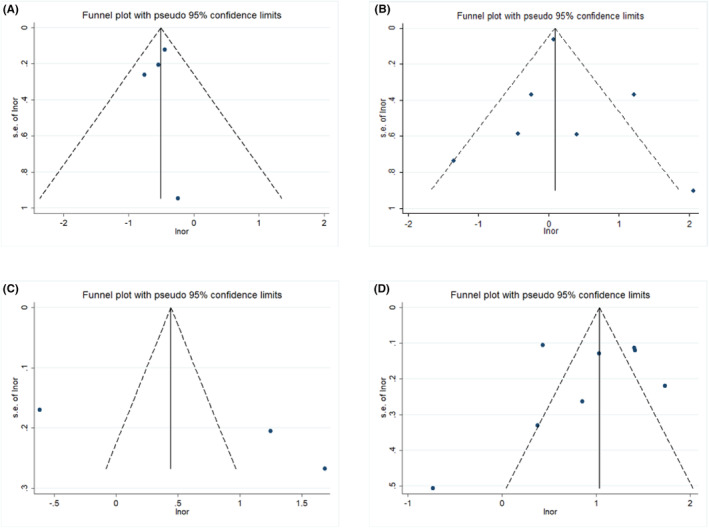
Funnel plots of the associations between four genetic variants and esophageal squamous cell carcinoma risk. (A) ALDH2 rs671, (B) ALDH2 rs674, (C) ADH1B rs1042026, and (D) ADH1B rs1229984.

No heterogeneity was found for the rs671 variant of ALDH2 (*I* ^2^ = 0, *p* = 0.821). However, high heterogeneity was observed for the rs674 (*I* ^2^ = 69.9), rs1229984 (*I* ^2^ = 91.3), and rs1042026 (*I* ^2^ = 96.4) variants (all *p* < 0.1) (Table 2). To find out the source of heterogeneity and due to the limited number of studies for rs1042026, we performed subgroup analyses for rs674 and rs1229984 based on publication year, the number of participants, and study country (Table S1). For both variants, the heterogeneity did not decrease substantially in the stratified subgroups defined above. When stratified by the number of study participants, rs1229984 was associated with a significantly increased risk of ESCC (OR: 2.91, 95% CI: 1.80–4.71) only in the studies with more than 1000 participants. In addition, significant positive associations were observed in Chinese (OR: 2.45, 95% CI: 1.53–3.90) and Japanese (OR: 2.74, 95% CI: 1.55–4.84) populations, in both case–control (OR: 2.29, 95% CI: 1.47–3.56) and genome‐wide association studies (OR: 4.10, 95% CI: 3.24–5.18), in the studies published before 2010 (OR: 2.84, 95% CI: 1.79–4.51) and after 2010 (OR: 2.14, 95% CI: 1.37–3.33). The meta‐regression showed that no variables could explain the source of the high heterogeneity between the included studies for the rs1229984 and rs674 (Tables S3 and S4). Sensitivity analysis showed that the results of the meta‐analyses were reliable (Figures S2–S5). Funnel plots, Begg's test, and Egger's test indicated no publication bias for the four variants (*p* > 0.1) (Figure 3 and Table S2).

### 3.4 Reliability of epidemiological evidence for significant associations

Table 2 also shows the results of the epidemiological evidence reliability for the four variants. For the amount of evidence, only the variant rs1229984 reached the A grade, and the other three variants (rs671, rs1042026, and rs674) reached the B grade. Regarding the replication of association, only rs671 achieved the A grade, whereas the other three variants (rs1042026, rs674, and rs1229984) reached only the C grade. All the four variants achieved the A grade for the protection from bias. By combining the three criteria, we found one variant (rs671) was classified as moderate evidence, and three variants (rs1042026, rs674, and rs1229984) were graded as weak evidence, respectively. In addition, the FPRP of rs671 and rs1229984 variants was less than 0.2, suggesting a true association, and the FPRPs of rs674 and rs1042026 were greater than 0.2, indicating a greater probability of false positive association. The BFDPs of rs671 and rs1229984 were less than 0.8, suggesting that a true association could be considered. However, both rs674 and rs1042026 had BFDP greater than 0.8, indicating that the association needs to be interpreted with caution. The results of FPRP and BFDP were consistent. Based on the results of Venice criteria, FPRP, and BDFP, the reliability of epidemiological evidence for the significant association between rs671 and the risk of ESCC was upgraded from moderate to strong, the association between rs1229984 and ESCC risk was upgraded from weak to moderate, and the evidence remained weak for rs1042026 and rs674.

### 3.5 Functional annotation

Functional annotation was assessed by the Encyclopedia of DNA Elements tool HaploReg v4.1 (Table 4).^27^ The rs671 and rs1229984 variants were located in exons, and the rs1042026 and rs674 variants were located in noncoding regions. The variants rs1042026 and rs674 were selected as expression quantitative trait loci (eQTL) hits, and the variants rs674, rs671, and rs1229984 were identified as enhancer histone marks. In addition, rs671, rs1042026, and rs674 were related to the motif change for some genes.

**TABLE 4:** Functional annotation for genetic variants that have high or moderate association with ESCC risk.

Variant	Gene	HaploReg v4.1										PolyPhen‐2	
		Promoter histone marks	Enhancer histone marks	DNase	Proteins bound	Motifs changed	NHGRI/EBI GWAS hits	GRASP QTL hits	Selected eQTL hits	RefSeq genes	dbSNP functional annotation	Predicted consequence on protein function	PolyPhen score
rs671	ALDH2	–	GI	–	–	4 altered motifs	12 hits	–	–	ALDH2	Missense	Possibly damaging	0.885
rs1042026	ADH1B	–	–	–	–	HNF4, Mef2, and Nr2f2	1 hits	–	4 hits	ADH1B	3’‐UTR	–	–
rs674	ALDH2	–	BLD, GI, BONE	4 tissues	–	Foxp1, NF‐I, ZEB1	–	–	9 hits	‐	3’‐UTR	–	–
rs1229984	ADH1B	GI, HRT	OVRY	–	–	–	4 hits	–	–	ADH1B	Missense	Benign	0.009

Abbreviations: ESCC, esophageal squamous cell carcinoma; −, data not available.

### 3.6 Mendelian randomization analysis

The results of the Mendelian randomization analysis showed that there was no causal effect between alcohol and esophageal cancer risk (OR: 0.99, 95% CI: 0.99–1.00) (Table 5). In the sensitivity analysis, the results using the weighted median and MR‐Egger methods were similar and did not reach statistical significance (*p* > 0.05). No heterogeneity was observed.

**TABLE 5:** Mendelian randomization analyses estimates for associations between alcohol and risk of ESCC.

Exposure	Outcome	SNPs, *n*	Methods	OR	95% CI	*p*‐Value
Alcohol	ESCC	5	IVW	0.99	0.99–1.00	0.71
		5	WM	0.99	0.99–1.00	0.88
		5	MR‐Egger	1.00	0.99–1.01	0.84

Abbreviations: CI, confidence interval; IVW, Inverse variance weighted; MR‐Egger, Mendelian randomization‐Egger; OR, odds ratio; WM, weighted median.

## 4 DISCUSSION

To our knowledge, this study is the first comprehensive assessment of the epidemiological evidence on the associations between genetic variants and ESCC risk in the ALDH2‐ADH1B region. We conducted a comprehensive research synopsis and meta‐analysis to evaluate the associations between the four variants and the risk of ESCC among 15,591 cases and 22,141 controls from different countries. We found that the polymorphism ADH1B rs1229984 was associated with an increased risk of ESCC. The evidence for the association between the rs671 variant and ESCC risk was considered as strong. Smokers with the rs1229984 risk genotype had further increased risk of ESCC compared with smokers without rs1229984 risk genotype. In addition, functional annotations of these variants identified that the variants were related to the enhancer histone marks and motif change. Individuals who carry risk alleles of ALDH2, ADH1B, or both should strengthen their health management. Necessary interventions could be performed in the prevention and screening of ESCC. For example, they should limit alcohol consumption and smoking,^28^ increase the intake of vegetables and fruits,^29^ and carry out regular gastroscopy screening.^30^, ^31^ Identification of the risk alleles of ALDH2 and ADH1B could help guide screening programs for the ESCC, thus reducing the incidence and mortality of ESCC for the high‐risk population.

A meta‐analysis that assessed the association between ADH1B and ALDH2 polymorphisms and esophageal cancer risk was published in 2010.^18^ Since then, 11 additional articles were published. We performed an updated analysis with the 11 new studies. In addition, we assessed the strength of the epidemiological evidence of the association using the Venice criteria, FPRP, and BDFP and found that the polymorphism rs671 showed strong evidence and rs1229984 had moderate evidence with ESCC risk, respectively.

Several genome‐wide association studies have identified a number of single nucleotide polymorphisms linked to the increased ESCC risk including ADH1B, PLCE1, genetic variants in HLA 2 genome region, CHEK2, PTEN, MTHFR, and so on. In a genome‐wide discovery, replication, and combined samples, eight genetic variants were identified as ESCC susceptibility variants, of which ADH1B had a significant interaction with ESCC risk (OR: 1.31) in drinker population. And the result of the case–control subgroup in the genome‐wide discovery was consistent with result of our meta‐analysis.^19^ The PLCE1 is the most notable one in the number of case–control studies, among which most studies found that PLCE1 was associated with increased ESCC risk.^32^, ^33^, ^34^ A joint analysis of three genome‐wide association studies indicated that Chinese populations with a variant in the HLA class II region had higher ESCC risk.^35^

For alcohol metabolism, a previous study showed that oxidation is catalyzed to produce acetaldehyde primarily by ADHs, and then acetaldehyde is further metabolized to acetate by ALDHs.^36^ Among the currently known genes, ALDH2 and ADH1B had the greatest impact on the risk of alcoholism, which can result in accumulation of acetaldehyde that causes DNA damage and promotes ESCC development.^37^, ^38^ ALDH2 is located on chromosome 12q24.2 and encodes the 517 amino‐acid aldehyde dehydrogenase 2 protein. We observed that the performance of rs671 in ESCC risk among the included studies is contradictory. This might be attributed to the differences in population, sample size, environmental exposure, and lifestyle. Our study found that the rs671 variant, in exon 12 of the gene, was associated with a 40% ESCC risk reduction, and the evidence was strong. The activity of enzyme encoded by the wild homozygous genotype GG of ALDH2 rs671 is normal, while the enzyme activity encoded by the heterozygous GA genotype is reduced but still has certain activity. The enzyme encoded by the mutant homozygous AA genotype is basically inactive, which resulted in the failure of acetaldehyde metabolism. A large accumulation of acetaldehyde can cause symptoms such as blushing and rapid heartbeat, which makes individuals carrying the ALDH2 rs671A allele with poor ability to metabolize alcohol. Therefore, the ESCC risk of individuals carrying the rs671A allele could be reduced. Yu et al. also found that the ALDH2 rs671 has a suppressive role in alcohol consumption, which was consistent with our results. In addition, tobacco smoke contains a high level of nicotine‐derived nitrosamine ketone and N‐nitrosonornicotine, which directly contact with the esophageal mucosa and further increase the risk esophageal cancer.^39^, ^40^ In this study, we found an interaction between rs671 and smoking, but it did not reach statistical significance, which had a difference with the result for the rs671 (Table 2). This might be attributed to the fact that some studies did not provide OR and 95% CI of the interaction, thus had reduced statistical power to detect the association. Similar results were also found in the study of Tanaka et al. (OR: 1.1, 95% CI: 0.5–2.4).^41^ In addition, several studies have reported that the variant is associated with the development of alcohol‐related cancers, including colorectal cancer and hepatocellular cancer.^42^, ^43^

ADH1B, mapped to 4q23, is about 15 kb in length, and contains nine exons, and can catalyze the rate of ethanol metabolization into acetaldehyde. The rs1229984, a non‐synonymous SNP in exon 3 of ADH1B, encodes lysine‐504 and is prone to T‐to‐C mutations, resulting in an Arg‐to‐His mutation. The genotype of ADH1B rs1229984 carrying allele T encodes a more active protein that can quickly metabolize ethanol into acetaldehyde, while the mutant homozygous CC genotype has very weak activity in metabolizing ethanol. It indicated that mutant homozygous CC genotype could increase ESCC risk. Our study found that this variant was associated with an increased risk of ESCC with moderate evidence. In 2022, a prospective study with 11 years of follow‐up of 9339 Chinese adults also observed that the rs1229984 AA genotype was associated with a lower risk of alcohol‐related cancers than the GG genotype among men.^44^ In addition, although the strength of the interaction between smokers (OR: 1.52) and nonsmokers (OR: 1.39) and rs1229984 was lower than rs1229984 with ESCC risk, smokers with the risk genotype had a further increased ESCC risk, suggesting an interaction between the genetic variant and smoking. Tanaka et al.^41^ also reported similar findings in the Japanese population. Besides, the largest GWAS of alcohol dependence revealed that ADH1B played an important role in the etiology of alcohol dependence among Europeans and African‐Americans. Considering that ADH1B rs1229984 is more common in the East Asian population than in Europeans,^45^, ^46^ more studies are needed to investigate the role of ADH1B in the etiology of alcohol dependence among the East Asian population. Several studies found that the ADH1B was associated with colorectal cancer and obesity,^47^, ^48^ suggesting its potential role in multiple diseases. In addition, although our results showed rs671 and 1,229,984 had a significant association with ESCC risk, it did not necessarily mean the causal effect of alcohol on the esophageal cancer by the Mendelian randomization analysis.^49^, ^50^

Due to the limited number of studies for ADH1B rs1042026, we only explored the source of the high heterogeneity for ALDH2 rs674 and ADH1B rs1229984. The included studies on these polymorphisms did not provide the ORs and the corresponding 95% CIs in the age and sex subgroups; therefore, the subgroup analyses were stratified on publication year, the number of participants, and study country. However, the high heterogeneity did not reduce dramatically after stratification. We did find that the Japanese population with the ADH1B rs1229984 risk genotype had a greater ESCC risk than the Chinese. Considering the interaction between the genotype and smoking observed in our study, the higher ESCC risk found in the Japanese population could be due to the higher prevalence of smoking in Japan.^51^ The magnitude of the association between ADH1B rs1229984 and ESCC risk attenuated slightly in the studies published after 2010, which could be due to the declining rate of alcohol and tobacco use and the widespread application of endoscopic screening.^52^, ^53^

This study has several limitations. First, most studies included were case–control studies, which are more susceptible to biases such as selection bias and information bias, particularly if the genotypes are associated with ESCC mortality. Secondly, some relevant articles had to be excluded due to missing data and no responses from the corresponding authors. Finally, because of the lack of raw data, we used crude ORs and 95% CIs, which will result in a slight deviation.

In summary, after including results from the 11 additional studies published after 2010, this updated meta‐analysis confirmed the associations between the genetic variants of ALDH2 rs671 and ADH1B rs1229984 and the ESCC risk, of which the association between rs671 and the ESCC risk was considered as strong evidence. An interaction between the genetic variant rs1229984 and smoking was also observed. More experiments regarding fundamental research are needed to further elaborate roles of these genetic variants in the development of ESCC in the future work.

## AUTHOR CONTRIBUTIONS

**Biao Zhang:** Resources (equal); software (equal); visualization (equal). **Yu;Hui Peng:** Funding acquisition (equal). **Yun Luo:** Resources (equal); writing – original draft (equal). **Chao Qun Hong:** Data curation (equal). **Yi Wei Lin:** Software (equal). **Yuling Zhang:** Visualization (equal). **Yi Wei Xu:** Writing – review and editing (equal). **Xuefen Su:** Methodology (equal); writing – review and editing (equal). **Fang Cai Wu:** Writing – review and editing (equal).

## FUNDING INFORMATION

This work was supported by the Natural Science Foundation of China [grant number 81972801]; the Guangdong Basic and Applied Basic Research Foundation Enterprise Joint Foundation (2022A1515220116, 2022A1515220180), the Science and Technology Special Fund of Guangdong Province of China (STKJ202209069), the Guangdong Medical Science and Technology Research Program (A2023414), the Guangdong Esophageal Cancer Institute Science and Technology Program (M202224).

## CONFLICT OF INTEREST STATEMENT

All authors declare no conflict of interest.

## Supporting information


Appendix S1

## Data Availability

All data generated or analyzed that were reported in this article will be available upon request.
