# Supplementary material for: Relationship between esophageal squamous cell carcinoma risk and alcohol‐related ALDH2 and ADH1B polymorphisms: Evidence from a meta‐analysis and Mendelian randomization analysis
Source: Cancer Med. 2023 Oct 5;12(20):20437–49. doi: 10.1002/cam4.6610 (PMC10652316; doi:10.1002/cam4.6610)
Supplement: Supplementary file 1 — Appendix S1 [file CAM4-12-20437-s001.docx]

| **Supplementary Table 1. The results of subgroup analysis for the ADH1B rs1229984 and ALDH2 rs674** | | | | | | |
| --- | --- | --- | --- | --- | --- | --- |
| **Variants** | **Variables** | **No of studies** | **Model** | **Pooled OR (95% CI)** | **Heterogeneity** | |
|  |  |  |  |  | **I^2^(%)** | P |
| **ADH1B rs1229984** | **Race** | 8 |  |  |  |  |
|  | China | 5 | Ran | 2.45 (1.53-3.90) | 88.6 | 0 |
|  | Japan | 3 | Ran | 2.74 (1.55-4.84) | 88.5 | 0 |
|  | **Design style** | 8 |  |  |  |  |
|  | case-control study | 7 | Ran | 2.29 (1.47-3.56) | 91.1 | 0 |
|  | Genome-wide association study | 1 | Ran | 4.10 (3.24-5.18) | - | - |
|  | **Participants number** | 8 |  |  |  |  |
|  | <1000 | 4 | Ran | 1.88 (0.77-4.61) | 88.7 | 0 |
|  | >1000 | 4 | Ran | 2.91 (1.80-4.71) | 94.4 | 0 |
|  | **Publication year** | 8 |  |  |  |  |
|  | Before 2010 | 5 | Ran | 2.84 (1.79-4.51) | 86.3 | 0 |
|  | After 2010 | 3 | Ran | 2.14 (1.37-3.33) | 85.1 | 0 |
| **ALDH2 rs674** | **Race** | 7 |  |  |  |  |
|  | China | 4 | Ran | 0.91 (0.32-2.59) | 79.5 | 0.002 |
|  | Japan | 3 | Ran | 1.67 (0.68-4.09) | 60.9 | 0.078 |
|  | **Participants number** | 7 |  |  |  |  |
|  | <1000 | 6 | Ran | 1.19 (0.64-2.22) | 74.5 | 0.001 |
|  | >1000 | 1 | Ran | 1.49 (0.47-4.75) | - | - |
|  | **Publication year** | 7 |  |  |  |  |
|  | Before 2010 | 5 | Ran | 1.05 (0.59-1.87) | 58.1 | 0.049 |
|  | After 2010 | 2 | Ran | 1.58 (0.32-7.90) | 82.4 | 0.017 |

Abbreviations: Ran: Random-effects model; OR: odds ratio; CI: confidence interval; -: unavailable data

| **Supplementary Table 2. The results of egger's test in meta-analysis** | | | | | | |
| --- | --- | --- | --- | --- | --- | --- |
| Variants | Std | t | *P* | 95% CI | |  |
| rs671 | 3.88 | -0.90 | 0.41 | -13.48 | 6.48 |  |
| rs1229984 | 3.01 | -0.54 | 0.61 | -8.99 | 8.73 |  |
| rs1042026 | 13.51 | 1.83 | 0.32 | -146.89 | 196.43 |  |
| rs674 | 0.95 | 0.42 | 0.69 | -2.03 | 2.83 |  |

| **Supplementary Table 3. The results of meta-regression for the rs1229984** | | | | | | |
| --- | --- | --- | --- | --- | --- | --- |
|  | Exp(b) | Std | t | p | 95% CI | |
| Race | 1.05 | 1.05 | 1.00 | 0.39 | -2.29 | 4.39 |
| publication year | 0.79 | .86 | 0.93 | 0.42 | -1.93 | 3.51 |
| Participants number | -0.85 | 0.204 | -0.9 | 0.379 | 0.461 | 1.363 |
| Design style | -0.59 | 1.17 | -0.50 | 0.65 | -4.30 | 3.13 |
| _cons | 0.62 | 1.23 | 0.50 | 0.65 | -3.29 | 4.53 |

| **Supplementary Table 4. The results of meta-regression for the rs674** | | | | | | |
| --- | --- | --- | --- | --- | --- | --- |
|  | Exp(b) | Std | t | p | 95% CI | |
| Race | -1.48 | 1.13 | -1.31 | 0.28 | -5.08 | 2.12 |
| publication year | 1.18 | 1.12 | 1.05 | 0.37 | -2.37 | 4.73 |
| Participants number | -0.37 | 1.40 | -0.27 | 0.81 | -4.83 | 4.09 |
| _cons | 0.77 | 0.80 | 0.97 | 0.41 | -1.76 | 3.31 |

**Supplementary Figures 1. Forest plots of the associations between the rs1229984(a), ALDH2 rs671(b) and esophageal squamous cell carcinoma risk in the allelic model.**

**
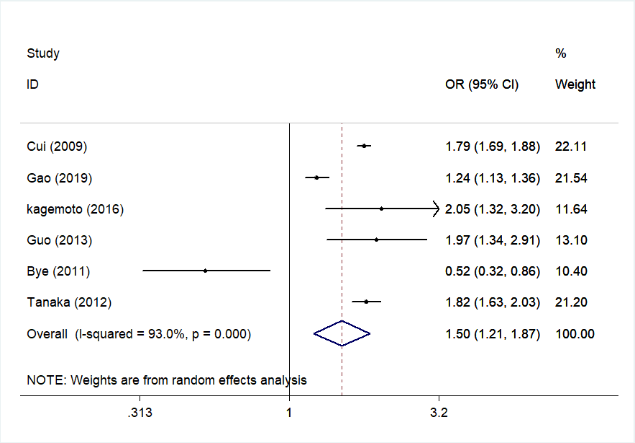

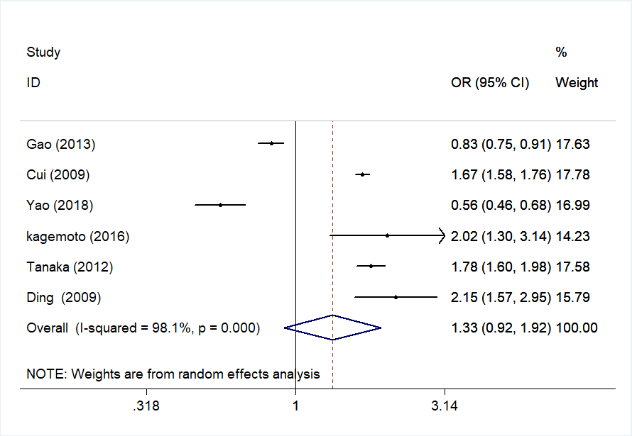
**

**a b**

**Supplementary Figures 2-5. Sensitivity analysis for the associations between the four genetic variants and esophageal squamous cell carcinoma risk. (1) ALDH2 rs671, (2) ALDH2 rs674, (3) ADH1B rs1042026, (4) ADH1B rs1229984.**


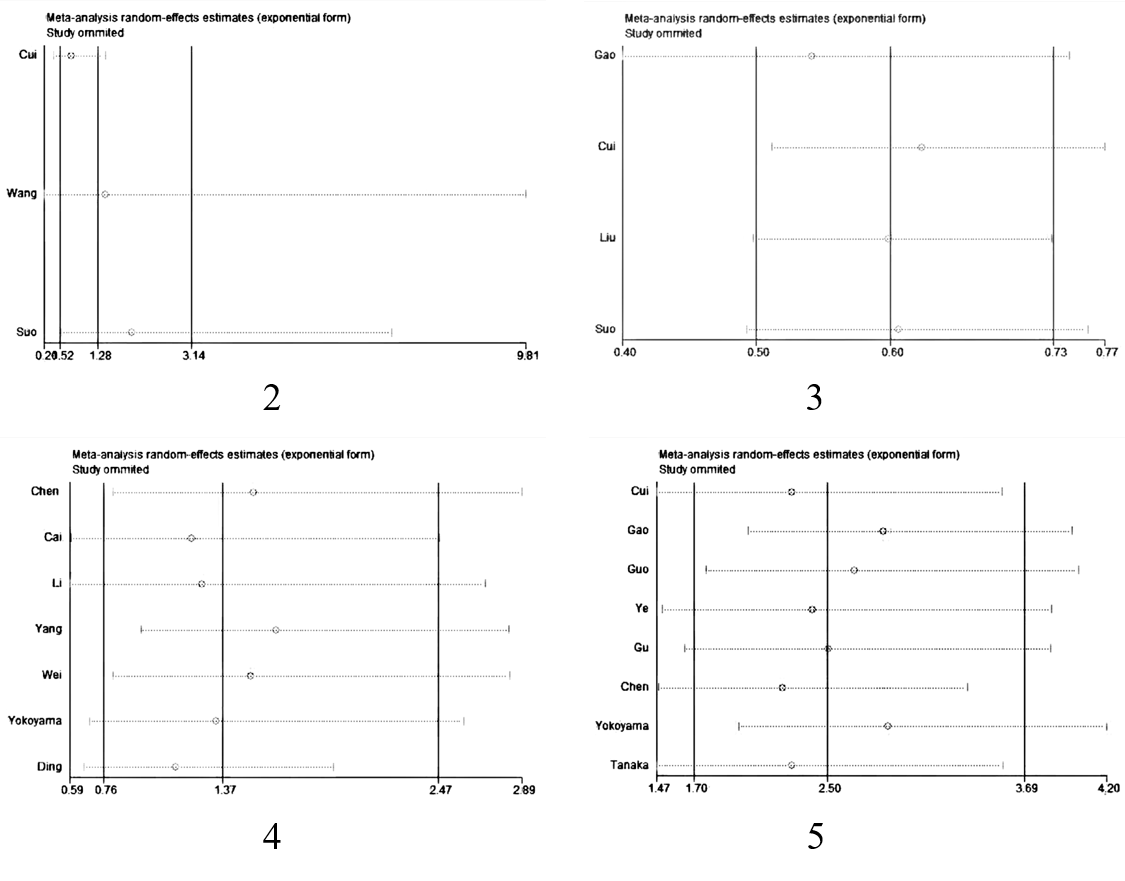


**Included publications**

1 Bye H, Prescott NJ, Matejcic M, Rose E, Lewis CM, Parker MI, Mathew CG. Population-specific genetic associations with oesophageal squamous cell carcinoma in South Africa. *Carcinogenesis* 2011; **32**(12): 1855-1861 [PMID: 21926110 PMCID: PMC3220606 DOI: 10.1093/carcin/bgr211]

2 Cai L, You NC, Lu H, Mu LN, Lu QY, Yu SZ, Le AD, Marshall J, Heber D, Zhang ZF. Dietary selenium intake, aldehyde dehydrogenase-2 and X-ray repair cross-complementing 1 genetic polymorphisms, and the risk of esophageal squamous cell carcinoma. *Cancer* 2006; **106**(11): 2345-2354 [PMID: 16639733 DOI: 10.1002/cncr.21881]

3 Chen WC, Bye H, Matejcic M, Amar A, Govender D, Khew YW, Beynon V, Kerr R, Singh E, Prescott NJ, Lewis CM, Babb de Villiers C, Parker MI, Mathew CG. Association of genetic variants in CHEK2 with oesophageal squamous cell carcinoma in the South African Black population. *Carcinogenesis* 2019; **40**(4): 513-520 [PMID: 30753320 PMCID: PMC6556703 DOI: 10.1093/carcin/bgz026]

4 Chen YJ, Chen C, Wu DC, Lee CH, Wu CI, Lee JM, Goan YG, Huang SP, Lin CC, Li TC, Chou YP, Wu MT. Interactive effects of lifetime alcohol consumption and alcohol and aldehyde dehydrogenase polymorphisms on esophageal cancer risks. *Int J Cancer* 2006; **119**(12): 2827-2831 [PMID: 17036331 DOI: 10.1002/ijc.22199]

5 Cui R, Kamatani Y, Takahashi A, Usami M, Hosono N, Kawaguchi T, Tsunoda T, Kamatani N, Kubo M, Nakamura Y, Matsuda K. Functional variants in ADH1B and ALDH2 coupled with alcohol and smoking synergistically enhance esophageal cancer risk. *Gastroenterology* 2009; **137**(5): 1768-1775 [PMID: 19698717 DOI: 10.1053/j.gastro.2009.07.070]

6 Ding JH, Li SP, Cao HX, Wu JZ, Gao CM, Su P, Liu YT, Zhou JN, Chang J, Yao GH. Polymorphisms of alcohol dehydrogenase-2 and aldehyde dehydrogenase-2 and esophageal cancer risk in Southeast Chinese males. *World J Gastroenterol* 2009; **15**(19): 2395-2400 [PMID: 19452585 PMCID: PMC2684609 DOI: 10.3748/wjg.15.2395]

7 Gao Y, He Y, Xu J, Xu L, Du J, Zhu C, Gu H, Ma H, Hu Z, Jin G, Chen X, Shen H. Genetic variants at 4q21, 4q23 and 12q24 are associated with esophageal squamous cell carcinoma risk in a Chinese population. *Hum Genet* 2013; **132**(6): 649-656 [PMID: 23430454 DOI: 10.1007/s00439-013-1276-5]

8 Gu H, Gong D, Ding G, Zhang W, Liu C, Jiang P, Chen S, Chen Y. A variant allele of ADH1B and ALDH2, is associated with the risk of esophageal cancer. *Exp Ther Med* 2012; **4**(1): 135-140 [PMID: 23060937 PMCID: PMC3460276 DOI: 10.3892/etm.2012.547]

9 Guo YM, Wang Q, Liu YZ, Chen HM, Qi Z, Guo QH. Genetic polymorphisms in cytochrome P4502E1, alcohol and aldehyde dehydrogenases and the risk of esophageal squamous cell carcinoma in Gansu Chinese males. *World J Gastroenterol* 2008; **14**(9): 1444-1449 [PMID: 18322963 PMCID: PMC2693697 DOI: 10.3748/wjg.14.1444]

10 Kagemoto K, Urabe Y, Miwata T, Oka S, Ochi H, Kitadai Y, Tanaka S, Chayama K. ADH1B and ALDH2 are associated with metachronous SCC after endoscopic submucosal dissection of esophageal squamous cell carcinoma. *Cancer Med* 2016; **5**(7): 1397-1404 [PMID: 27038040 PMCID: PMC4944865 DOI: 10.1002/cam4.705]

11 Li QD, Li H, Wang MS, Diao TY, Zhou ZY, Fang QX, Yang FY, Li QH. Multi-susceptibility genes associated with the risk of the development stages of esophageal squamous cell cancer in Feicheng County. *BMC Gastroenterol* 2011; **11**: 74 [PMID: 21672255 PMCID: PMC3141752 DOI: 10.1186/1471-230x-11-74]

12 Liu P, Zhao HR, Li F, Zhang L, Zhang H, Wang WR, Mao R, Su WP, Zhang Y, Bao YX. Correlations of ALDH2 rs671 and C12orf30 rs4767364 polymorphisms with increased risk and prognosis of esophageal squamous cell carcinoma in the Kazak and Han populations in Xinjiang province. *J Clin Lab Anal* 2018; **32**(2) [PMID: 28464297 PMCID: PMC6816997 DOI: 10.1002/jcla.22248]

13 Ma WJ, Lv GD, Zheng ST, Huang CG, Liu Q, Wang X, Lin RY, Sheyhidin I, Lu XM. DNA polymorphism and risk of esophageal squamous cell carcinoma in a population of North Xinjiang, China. *World J Gastroenterol* 2010; **16**(5): 641-647 [PMID: 20128036 PMCID: PMC2816280 DOI: 10.3748/wjg.v16.i5.641]

14 Suo C, Yang Y, Yuan Z, Zhang T, Yang X, Qing T, Gao P, Shi L, Fan M, Cheng H, Lu M, Jin L, Chen X, Ye W. Alcohol Intake Interacts with Functional Genetic Polymorphisms of Aldehyde Dehydrogenase (ALDH2) and Alcohol Dehydrogenase (ADH) to Increase Esophageal Squamous Cell Cancer Risk. *J Thorac Oncol* 2019; **14**(4): 712-725 [PMID: 30639619 DOI: 10.1016/j.jtho.2018.12.023]

15 Tanaka F, Yamamoto K, Suzuki S, Inoue H, Tsurumaru M, Kajiyama Y, Kato H, Igaki H, Furuta K, Fujita H, Tanaka T, Tanaka Y, Kawashima Y, Natsugoe S, Setoyama T, Tokudome S, Mimori K, Haraguchi N, Ishii H, Mori M. Strong interaction between the effects of alcohol consumption and smoking on oesophageal squamous cell carcinoma among individuals with ADH1B and/or ALDH2 risk alleles. *Gut* 2010; **59**(11): 1457-1464 [PMID: 20833657 DOI: 10.1136/gut.2009.205724]

16 Wang J, Wei J, Xu X, Pan W, Ge Y, Zhou C, Liu C, Gao J, Yang M, Mao W. Replication study of ESCC susceptibility genetic polymorphisms locating in the ADH1B-ADH1C-ADH7 cluster identified by GWAS. *PLoS One* 2014; **9**(4): e94096 [PMID: 24722735 PMCID: PMC3983154 DOI: 10.1371/journal.pone.0094096]

17 Wang Y, Ji R, Wei X, Gu L, Chen L, Rong Y, Wang R, Zhang Z, Liu B, Xia S. Esophageal squamous cell carcinoma and ALDH2 and ADH1B polymorphisms in Chinese females. *Asian Pac J Cancer Prev* 2011; **12**(8): 2065-2068 [PMID: 22292652]

18 Yang SJ, Wang HY, Li XQ, Du HZ, Zheng CJ, Chen HG, Mu XY, Yang CX. Genetic polymorphisms of ADH2 and ALDH2 association with esophageal cancer risk in southwest China. *World J Gastroenterol* 2007; **13**(43): 5760-5764 [PMID: 17963305 PMCID: PMC4171265 DOI: 10.3748/wjg.v13.i43.5760]

19 Yao L, Yu F, Mao Y, Wang T, Qi Q, Ding H, Wang J, Ma H, Dai J, Zhang G, Jin G. Gastric cancer may share genetic predisposition with esophageal squamous cell carcinoma in Chinese populations. *J Hum Genet* 2018; **63**(11): 1159-1168 [PMID: 30202044 DOI: 10.1038/s10038-018-0501-4]

20 Ye B, Feng J, Pan X, Yang Y, Ji C, Cheng M, Cheng Y, Shi J, Zhao H. Genetic variant of single-nucleotide polymorphism is associated with risk of esophageal squamous cell carcinoma. *Genet Test Mol Biomarkers* 2014; **18**(1): 45-49 [PMID: 24093763 DOI: 10.1089/gtmb.2013.0336]

21 Yokoyama A, Kato H, Yokoyama T, Igaki H, Tsujinaka T, Muto M, Omori T, Kumagai Y, Yokoyama M, Watanabe H. Esophageal squamous cell carcinoma and aldehyde dehydrogenase-2 genotypes in Japanese females. *Alcohol Clin Exp Res* 2006; **30**(3): 491-500 [PMID: 16499490 DOI: 10.1111/j.1530-0277.2006.00053.x]

22 Yokoyama A, Kato H, Yokoyama T, Tsujinaka T, Muto M, Omori T, Haneda T, Kumagai Y, Igaki H, Yokoyama M, Watanabe H, Fukuda H, Yoshimizu H. Genetic polymorphisms of alcohol and aldehyde dehydrogenases and glutathione S-transferase M1 and drinking, smoking, and diet in Japanese men with esophageal squamous cell carcinoma. *Carcinogenesis* 2002; **23**(11): 1851-1859 [PMID: 12419833 DOI: 10.1093/carcin/23.11.1851]

23 Yokoyama A, Yokoyama T, Muramatsu T, Omori T, Matsushita S, Higuchi S, Maruyama K, Ishii H. Macrocytosis, a new predictor for esophageal squamous cell carcinoma in Japanese alcoholic men. *Carcinogenesis* 2003; **24**(11): 1773-1778 [PMID: 12949054 DOI: 10.1093/carcin/bgg142]
